# Plant Production Protocols from Seeds of Threatened *Atropa baetica* and Widespread *A. belladonna*, Both Rich in Alkaloids

**DOI:** 10.3390/life13112181

**Published:** 2023-11-08

**Authors:** Elena Copete, Miguel A. Copete, Esmeralda Martínez-Duro, Alejandro Santiago, Pablo Ferrandis, José M. Herranz

**Affiliations:** 1 ETSIAMB, Department of Plant Production and Agricultural Technology, University of Castilla-La Mancha, University Campus s/n, 02071 Albacete, Spain; mariaelena.copete@uclm.es (E.C.); esmeralda.martinezduro@gmail.com (E.M.-D.); pablo.ferrandis@uclm.es (P.F.); jose.herranz@uclm.es (J.M.H.); 2 Botanical Institute, University of Castilla-La Mancha, Avenida de la Mancha s/n, 02006 Albacete, Spain; conservador@jardinbotanico-clm.com

**Keywords:** *Atropa baetica* Willk., *Atropa belladonna* L., dormancy breakage, germination, nondeep physiological dormancy, Solanaceae, tropane alkaloid

## Abstract

Members of the genus *Atropa* contain various tropane alkaloids, including atropine ((±)-hyoscyamine) and scopolamine, which possess medicinal properties. Preserving the diverse genetic background of wild populations via optimal plant production from seeds could be essential for avoiding the loss of potential uses. We analyzed the germination ecology of two *Atropa* species comprising the threatened *A. baetica* and widespread *A. belladonna* to determine the: (1) influence of temperature, light, and seed age on germination patterns; (2) effects of cold stratification and gibberellic acid (GA_3_); (3) phenology of seedling emergence in outdoor conditions; (4) phenology of dormancy break and loss of viability in buried seeds; and (5) ability to form persistent soil seed banks. Freshly matured seeds exhibited conditional physiological dormancy, with germination at high temperatures (32/18 °C) but not at low and cold ones (5, 15/4, 20/7 °C). The germination ability increased with time of dry storage and with GA_3_, thereby suggesting nondeep physiological dormancy. Under outdoor conditions, no seedlings emerged during the first post-sown autumn, but emergence peaks occurred in late winter–early spring. Both species could form small persistent soil seed banks with short durations (3–5 years). A plant production protocol from seeds was established for both taxa.

## 1. Introduction

Many plants have crucial roles as suppliers of resources for humans from secondary metabolites because some are extracted for the biotechnological production of compounds with medicinal aims. In particular, tropane alkaloids are the most important naturally occurring antimuscarinic drugs and they are found specifically in some Solanaceae genera, such as *Atropa*, *Anisodus*, *Datura*, *Duboisia*, and *Hyoscyamus*. Fifteen different tropane alkaloids and derivatives were identified in *Atropa baetica* Willk. [1] and *Atropa belladonna* L. [2]. In fact, *A. belladonna* is one of the most important herbal plants that produce hyoscyamine or atropine, and it also produces anisodamine and scopolamine [3].

Advances in the field of biotechnology have facilitated the in vitro synthesis and production of plant secondary metabolites from plant cell and tissue cultures under sterile conditions [4]. In particular, root cultures are derived from various Solanaceae species to obtain atropine, scopolamine, and hyoscyamine because the major storage site of these chemicals is in the main root [5]. However, secondary metabolites are modulated by various factors, including physiological, genotypic, and environmental factors [6]. Thus, biotechnological industries must recognize that valuable metabolites are genetically associated, and the production of some could be lost if only specific clones obtained from in vitro culture are used. These secondary metabolite compounds are biosynthesized via specific different pathways for each class of chemicals produced in plants, where the modulation of a single gene influences the production and accumulation of metabolites. Thus, preserving the diverse genetic background of wild populations by plant production from seeds could be essential for future commercial and pharmacological applications in aroma chemical and drug industries.

Consequently, optimizing plant production is a fundamental tool for extracting metabolites from lines of individuals with wide genetic backgrounds [7]. Thus, the main aim of the present study was to identify optimal protocols for obtaining plants from *Atropa* seeds using an efficient method. In particular, we focused on two species that produce tropane alkaloids comprising the threatened *A. baetica* and widespread *A. belladonna*. The selection of these two species from the same genus but with marked differences in the sizes of their distribution areas allowed us to compare the germination requirements and to analyze their use as sources of metabolites without affecting the conservation of natural populations, especially in the case of *A. baetica*. A second aim of this study was to analyze whether the germination ecology may result in a bottleneck responsible for the threatened status of species by comparing *A. baetica* with *A. belladonna*. Many comparative studies have investigated rare and widely distributed species with close phylogenetic relationships, especially those that belong to the same genus, usually by characterizing the life traits related to phenological or reproductive aspects, such as the production of flowers, fruits, and seeds, reproductive success, and seed germination in order to determine the causes of rarity [8,9,10].

The timing of seed germination is a complicated process in some plant species, and it is a highly adaptive trait that helps to increase the likelihood of a plant completing its life cycle [11]. In strongly seasonal environments, germination regulation aims to maximize the probability of seedling establishment and the perpetuation of plants [12]. Frequently, regulation involves dormancy that can be ended by environmental cues that cause seeds to germinate before optimal conditions for establishment [13]. Thus, in many temperate region species, seeds are produced in the summer or fall, and they possess mechanisms for delaying germination until the following spring. These mechanisms function in a predictable manner to prevent precocious germination in the fall and winter. The seeds of populations in sites with severe winters are less likely to germinate under autumn or winter conditions than those of populations in sites with mild winters because of the increased risk associated with severe winters. Rapid germination will occur the following spring if obstacles to germination, such as dormancy, are removed by winter chilling [14].

A second function of germination regulation is to prevent total germination before optimal conditions for establishment, thereby ensuring the carryover of seeds. In species that form persistent seed banks, a fraction of seeds may respond to environmental cues during the first year but the remainder resist these cues and carry over as viable, ungerminated seeds from year to year [12,15]. One of the aims of the present study was to determine the importance of these functions for germination regulation in two species comprising *A. belladonna* and *A. baetica*. Similarly, studying persistent soil seed banks is crucial for species with special protection habitats (*A. belladonna*) or endangered species (*A. baetica*) because it increases the probability of population re-establishment after a disturbance without external propagule inputs [16,17], and seed banks may confer ecological advantages in the context of population dynamics [18].

It is also known that variations in the germination requirements between species and populations of a certain species may represent adaptations to the local habitat conditions, where these differences are determined genetically [19]. A previous study of 32 temperate *Carex* species [20] found that differences in the germination requirements were related to habitat preferences. Dormancy is also frequently related to altitude because populations at high altitudes that spend prolonged periods under snow cover require longer periods of cold stratification [21]. Thus, another aim of the present study was to verify whether the germination ecology reflected strong adaptations to the habitats of the two target species. Therefore, the germination capacity was analyzed for seeds with different ages after cold stratification, followed by exposure to a wide range of temperature and light conditions, which simulated the natural environmental cues throughout the year. The effects of dry storage duration and gibberellic acid (GA_3_) on germination were analyzed to determine the level of physiological seed dormancy [22].

*Atropa baetica* is a perennial herbaceous rhizomatous plant, which is endemic to the center and south of the Iberian Peninsula and North Africa. Due to its scarce and reduced populations, this species is included in the “in extinction danger” category in the Spanish catalogue of endangered species and in the Red list of Spanish vascular flora [23]. *Atropa baetica* is also included in Annex II of the Habitat Directive of the European Union as a priority conservation species. The recovery plan for this species [24] recommends reinforcement programs through the production of ex situ plants.

*Atropa belladonna* is also a perennial herbaceous rhizomatous, but it has a wide geographical distribution in the center and south of Europe, West Asia, and North Africa [25]. *Atropa belladonna* is not threatened but it grows in priority conservation concern habitats, such as pine forest with *Pinus nigra* subsp. *salzmannii* and deciduous Euro-Siberian communities (forests of *Tilia platyphyllos*, *Populus tremula*, *Betula pendula*, and *Corylus avellana*), which are protected conservation habitats in a favorable conservation state and population reinforcement programs may be required for the species present in them [26].

The specific aims of the present study of the germination ecology of *A. baetica* and *A. belladonna* to identify detailed optimal plant production protocols were: (1) to test the effects of light and temperature on seed germination and analyze the influence of the duration of seed dry storage; (2) to assess the effects of GA_3_ and cold stratification on seed germination; (3) to characterize the phenology of seedling emergence in shadehouse conditions; (4) to determine the phenology of dormancy breaking using buried seeds that we recovered periodically; and (5) to determine the capacity of both species to form persistent soil seeds banks.

## 2. Materials and Methods

### 2.1. Plant Materials and Seed Sources

- *Atropa baetica*

Adult *A. baetica* individuals have a robust rhizome, which is often branched, and reach a height of up to 60–80 cm and diameter of 2–4 cm (personal observation). The rhizome produces up to 15–20 straight stems measuring 90–160 cm and each has 30–50 flowers, with funnel-shaped, gamopetalous, yellow corollas that measure 2–3 cm in length. The flowers are hermaphrodite with generalist entomogamous pollination. Flowering occurs in June–July and 90–95% of the flowers produce fruits that ripen in August–early September. The fruits are black berries with a diameter of 10–15 mm and they contain 20–30 reniform, brownish seeds with dimensions of 2–3 × 2 mm. *A. baetica* grows on the edges of forest trails and forest clearings (pine forests, gall oaks, holm oaks, etc.) over limestone soils between 900 and 2000 m a.s.l., where this species forms small population centers (2–6 individuals) separated by several kilometers. In each of the population centers, vegetative reproduction from vigorous new rhizomes is predominant, and few seedlings can overcome the competition with shoots and other vegetation. However, sexual reproduction plays a crucial role in the establishment of new populations from seeds dispersed by birds after eating berries [23,25].

In the present study, seeds were collected from plants growing in the Botanical Garden of Castilla-La Mancha, Albacete, Spain, to avoid affecting the dispersal of natural populations. These plants were produced from seeds collected in the Hosquillo hunting park (Las Majadas, Cuenca province, 30TWK8868, 1560 m), which contains a very vigorous population center but with low production of viable seeds (<2%). On 12 August 2016, 500 ripe berries were collected from five adult individuals aged over 4 years. Berries were macerated in the laboratory under running cold water to obtain a seed/pulp mixture, before drying for 48 h and separating the seeds by using a series of sieves. Dried seeds were spread on trays until 1 September. At this point (seed age = 0 month), seeds were stored in paper envelopes at room temperature in the laboratory (22–24 °C, relative humidity = 40–50%) until the experiments commenced.

- *Atropa belladonna*

*Atropa belladonna* is very similar morphologically to its congeners but with purple flowers instead of yellow. The rhizomes are less robust than those of *A. baetica* and it produces a smaller number of stems and flowers. The phenological cycle is similar to that of *A. baetica*. The fruits are black berries and thicker than those in other species (diameter of 13–18 mm). The fruit contains 25–35 dark brown, reniform seeds, which are slightly smaller (1.5–2 × 1.5 mm) than those produced by *A. baetica*. Both taxa grow in similar habitats, and they can even share the same area and hybridize. *Atropa belladonna* frequently has very large populations [25].

The seeds used in this study were collected in the exploratory hunting park called El Hosquillo (located in Las Majadas, Cuenca province, 30TWK8968, 1240 m) from clearings in a *Pinus nigra* subsp. *salzmannii* pine forest located at a slightly lower altitude than that for *A. baetica*. The *A. belladonna* population comprised several hundred individuals. On 18 August 2016, 600 ripe berries were collected from about 30 plants. After collection, the seeds were processed using the same method applied to the seeds of *A. baetica*.

### 2.2. Laboratory Experiments

#### 2.2.1. General Conditions for Germination Experiments

Experiments were carried out within environmentally controlled chambers (Ibercex mod. F4, Spain) equipped with a digital control system for temperature (±0.1 °C) and lighting conditions (utilizing cool white fluorescent light at a flux density of 25 μmol m^−2^ s^−1^, equivalent to 1350 lux). Radicle emergence was determined in seeds under a daily photoperiod of 12 h (light) and under continuous darkness (darkness), which was achieved by wrapping Petri dishes in a double layer of aluminum foil, at a constant temperature of 5 °C and at 12/12 h daily fluctuating temperature regimes of 15/4 °C, 20/7 °C, 25/10 °C, 28/14 °C, and 32/18 °C, thereby yielding 12 light × temperature treatments. In the 12/12 h alternating temperature treatments, the higher temperature coincided with the light phase and the lower temperature with darkness. Each treatment used 100 seeds, which were distributed in four replicates each with 25 seeds. Each replicate was incubated in a Petri dish with a diameter of 9 cm on two layers of filter paper moistened with distilled water. Dishes were sealed with Parafilm to minimize the loss of water. Tests lasted 30 days. In treatments under light, the seeds were checked to assess germination every 2–3 days. Seeds incubated in darkness were checked only at the end of the tests.

The alternate temperature treatments replicated the average maximum and minimum monthly temperatures throughout the annual climatic cycle in continental regions within the Iberian Peninsula. Specifically, 15/4 °C represented November and March, 20/7 °C for October and April, 25/10 °C for September and May, 28/14 °C for June and August, and 32/18 °C for July. The average temperature observed in winter (December, January, and February) was simulated with the 5 °C treatment [27].

A seed was considered germinated when it exhibited a clearly visible radicle (≥1 mm). The germination percentage was determined based on the number of seemingly viable seeds. The viability of seeds that did not germinate was evaluated by examining the embryo’s characteristics, notably its color and turgidity. Seeds were classified as viable when the embryo exhibited a white coloration and withstood slight pressure when tweezers were applied. These criteria for seed viability closely correlated with the outcomes of the tetrazolium test. The germination velocity was tested with the T50 parameter defined as the time required to reach 50% of the final germination level [28].

#### 2.2.2. Effects of Light and Temperature, and Seed Storage Time on Seed Germination

For each species, three groups each containing 1200 seeds were used to test germination at the following different seed ages: 0 months (beginning on 1 September 2016), 4 months (beginning on 2 January 2017), and 8 months (beginning on 1 May 2017). On these dates, for each species, a subset containing 100 seeds was incubated for each of the 12 light × temperature treatments according to the method described in the previous section.

#### 2.2.3. Effects of Cold Stratification on Seed Germination

This experiment tested whether exposing seeds to cold during the winter months stimulated subsequent germination during spring compared with seeds that did not undergo cold stratification. The cold stratification period was 2 months, which is usual in the natural habitats of the target species (at the center of the Iberian Peninsula).

On 1 December 2016, for each species, one set of 1250 seeds was placed on two sheets of wet filter paper in a Petri dish with a diameter of 16 cm, and kept at 5 °C for 2 months. After the stratification period, subgroups of 100 seeds were incubated according to the 12 light × temperature treatments described above.

#### 2.2.4. Effects of GA_3_ on Seed Germination

The level of physiological dormancy is determined by the effect of GA_3_ on germination. On 1 May 2017 (seed age = 8 months), 100 seeds from each species were distributed into four replicates of 25 seed and placed on two sheets of filter paper, which were moistened with a solution containing 2000 ppm GA_3_ [29,30], before incubating according to the 12 light × temperature treatments for 30 days. The seed germination results were compared with those obtained without GA_3_ treatment for seeds aged 8 months and incubated under the same conditions, where distilled water was used instead of the GA_3_ solution.

### 2.3. Outdoor Experiments

These experiments were conducted with the goal of determining the timing of significant events during the seed and seedling stages in the life cycles of *A. baetica* and *A. belladonna* in relation to the cyclic temperature patterns throughout the year. Seeds were exposed to conditions closely resembling natural temperature variations within a nonheated shadehouse situated in Albacete at 690 m a.s.l. (central Spain, 150 km from El Hosquillo). The shadehouse’s air temperature underwent continuous recording via a data logger to evaluate the average monthly maximum and minimum temperatures.

The growth substrate utilized within the containers, where the seeds were housed, consisted of a combination of sterilized peat and sand (2:1 *v*/*v*). To replicate the natural soil moisture levels, the irrigation system was pre-set to reach field capacity on a weekly basis, with a bimonthly reduction during July and August to emulate the typical summer aridity of the Mediterranean region. Moreover, irrigation was suspended during the winter season when the substrate was frozen.

#### 2.3.1. Phenology of Seedling Emergence

On 1 September 2016, for each species, three trays (20 × 30 × 8 cm) with a drainage system were filled with substrate. Seeds were sown equidistantly at a depth of 3–4 mm. Three replicate trays were monitored weekly for each species. The emergence of seedlings was recorded, and they were subsequently removed.

#### 2.3.2. Phenology of Dormancy Break and Radicle Emergence

On 1 September 2016, groups of 100 seeds aged 0 months were mixed with fine-grained sterilized sand in a fine-mesh polyester cloth bag. Six bags containing *A. baetica* seeds were buried at a depth of 5 cm in a pot with sand and six other bags containing *A. belladonna* seeds were buried in another pot. Both pots were placed in the shadehouse. The bags were removed every 6 months starting on 1 March 2017, and the contents were sieved (1 mm) to separate the seeds from sand. The seeds with emerged radicles were counted and the nongerminated seeds were incubated for 30 days at 28/14 °C in the light. After the incubation period, the following percentages were used to assess the condition of the seeds: (1) seeds with emerged radicles in bags, (2) viable nondormant seeds (i.e., those that germinated during incubation at 25/10 °C), (3) viable dormant seeds (i.e., those that did not germinate at 25/10 °C, but had healthy embryos), and (4) nonviable seeds (i.e., those displaying signs of decay, lacking embryos, or containing deceased embryos when excised).

#### 2.3.3. Analysis of the Soil Seed Bank

*Atropa baetica* and *A. belladonna* seed banks were assessed by analyzing the seed contents of soil samples collected in areas located at hunting sites in Hosquillo (Las Majadas, Cuenca). Soil samples were collected on 1 July 2017, after autumn–winter–spring germination and before the start of seed dispersal from fruits produced that year. This sampling date was determined based on the concept of a persistent seed bank [31] in order to quantify the persistent fraction of the seed bank overlapping two consecutive phenological cycles.

For each species, four soil samples with dimensions of 20 × 20 × 5 cm were extracted and preserved in four plastic bags. In the laboratory, the samples were dried completely, and organic remains and coarse particles were separated by using a sieve with a mesh size of 5 mm. The seed content was assessed by using the physical separation method [32]. The soil was washed through a sieve with a mesh size of 1 mm and the seeds were separated under a binocular microscope.

Most of the seeds recovered from each soil sample were empty (nonviable) because they did not resist soft pressure applied with steel tweezers. To determine the proportion of viable seeds, full seeds were incubated at 28/14 °C in the light for 30 days. Germinated seeds were then counted as viable, as well as ungerminated seeds with embryos that had a healthy appearance, particularly in terms of their turgidity and color.

### 2.4. Statistical Analyses

For each species, we computed means and standard errors for the percentages of radicle and shoot emergences. The variables examined included stratification temperature, duration of stratification, incubation temperature, light condition during stratification–incubation, GA_3_ concentration (0 and 2000 ppm), and seed age. For each trial, the effects of multiple factors on germination were assessed through a multifactorial analysis of variance using SPSS Statistics v24. Seed germination capacity was determined based on the final cumulative germination percentage by the number of viable seeds. If a factor demonstrated significance, differences were further examined with Tukey’s multiple comparison test. Data normality (Cochran test) and homoscedasticity (David test) were assessed before conducting the analyses. The final cumulative germination percentages were subjected to a square root arcsine transformation.

## 3. Results

### 3.1. Laboratory Experiments

#### 3.1.1. Effects of Light and Temperature, and Seed Storage Time on Seed Germination

- *Atropa baetica*

**Figure 1:**
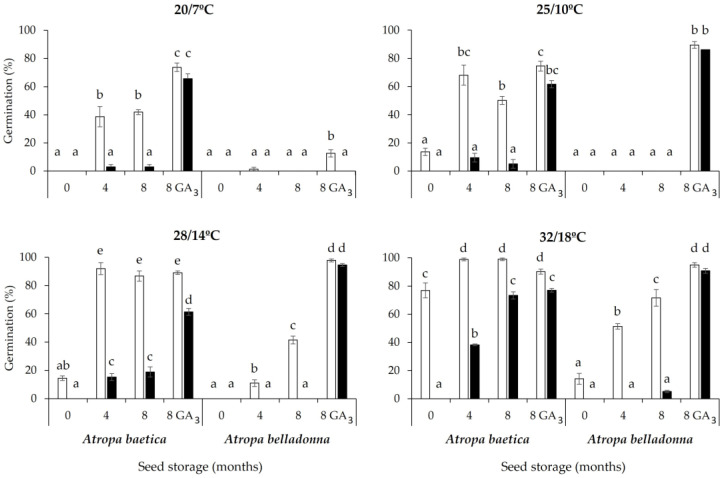
Germination of *Atropa baetica* and *A. belladonna* seeds after three different seed storage periods of 0, 4, and 8 months. Gibberellic acid (GA_3_ 2000 ppm) was applied to seeds aged 8 months. Results are shown after incubation at different thermoperiods (20/7 °C, 25/10 °C, 28/14 °C, and 32/18 °C). White and black bars indicate light and dark incubation conditions, respectively. No germination occurred at 5 °C and 15/4 °C. Different letters for each thermoperiod and species indicate significant differences (*p* < 0.05).

The germination percentage increased with the seed storage time (F_3,120_ = 74.5; *p* < 0.0001) and incubation temperature (F_3,120_ = 38.4; *p* < 0.0001), and under light conditions (F_1,120_ = 154.7; *p* < 0.0001). No germination occurred at 5 °C and 15/4 °C (Figure 1). Thus, seeds aged 0 months did not germinate at 20/7 °C but 77% germinated at 32/18 °C under light, and no new seeds germinated at any temperature under darkness. However, 42% of seeds aged 8 months germinated at 20/7 °C under light, and 73% at 32/18 °C under darkness. The germination rate was slow and influenced more by the incubation temperature than seed age. Thus, the T50 values were 26, 20, and 17.5 days for seeds incubated under light at 20/7 °C, 28/14 °C, and 32/18 °C, respectively.

- *Atropa belladonna*

The germination percentages were much lower for *A. belladonna* than those for *A. baetica* (F_1,247_ = 70.2; *p* < 0.0001). However, the trends were similar in this species with increased germination at higher incubation temperatures (F_3,120_ = 23.8; *p* < 0.0001) and seed ages (F_3,120_ = 86.6; *p* < 0.0001), and with incubation under light conditions (F_1,120_ = 14.6; *p* = 0.0002) (Figure 1). Seeds aged 0 months did not germinate at any of the temperatures tested. Very few seeds aged 4 months and 8 months germinated at 20/7 °C and 25/10 °C. The highest germination percentages were obtained for seeds aged 8 months incubated at 32/18 °C, with 72% under light but only 5% under darkness. The rate of germination was very slow depending on the incubation temperature. For seeds aged 8 months, the T50 values were 26 days at 28/14 °C and 24 days at 32/18 °C.

#### 3.1.2. Effects of Cold Stratification on Seed Germination

- *Atropa baetica*

**Figure 2:**
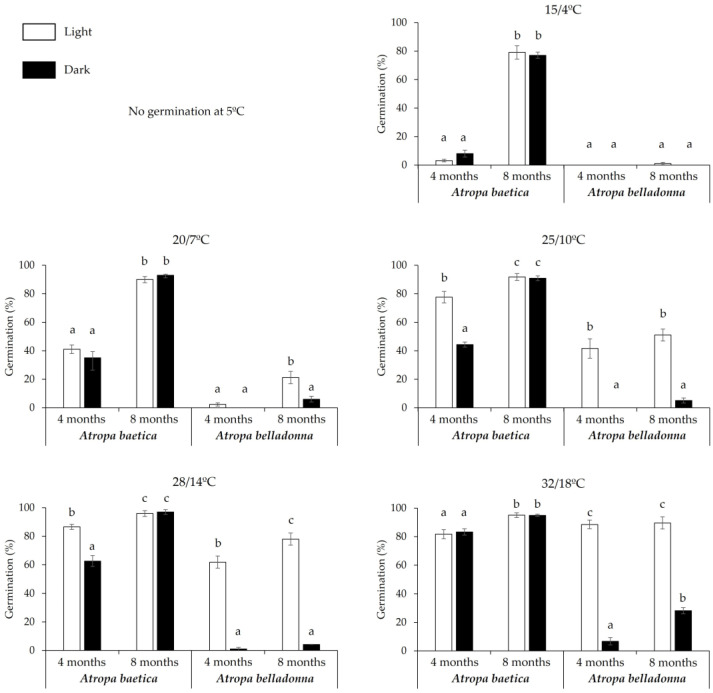
Seed germination after 2 months of cold stratification for seeds of *Atropa baetica* and *A. belladonna* previously stored under laboratory conditions for 4 months or 8 months. Results are shown after incubation at different thermoperiods (15/4 °C, 20/7 °C, 25/10 °C, 28/14 °C, and 32/18 °C) and at a constant temperature of 5 °C. Different letters for each thermoperiod and species indicate significant differences (*p* < 0.05).

Cold stratification at 5 °C for 2 months increased the germination percentages compared with nonstratified seeds at all temperatures and illumination conditions, except seeds incubated at 5 °C which did not germinate. Germination increased at 15/4 °C and 20/7 °C, which are typical temperatures at the end of winter and beginning of spring and they are favorable for germination from an ecological perspective (Figure 2).

In seeds with a similar age (4 months), nonstratified seeds did not germinate at 15/4 °C, but low germination occurred if they were previously cold stratified for 2 months. This effect was most obvious at 20/7 °C under darkness, where the germination rates varied between 3% and 35% without or with cold stratification, respectively (Figure 1 and Figure 2).

- *Atropa belladonna*

In this species, cold stratification stimulated germination at warm incubation temperatures. After storage for 4 months, 42% of the seeds germinated at 25/10 °C under light following cold stratification, but no germination occurred without stratification (Figure 1 and Figure 2).

#### 3.1.3. Effects of GA_3_ on Seed Germination

- *Atropa baetica*

GA_3_ promoted germination at 20/7 °C, 25/10 °C, and 28/14 °C under light and dark compared with untreated seeds. The increases in germination were more obvious under darkness, with increases from 3% to 65% at 20/7 °C, from 5% to 62% at 25/10 °C, and from 19% to 61% at 28/14 °C. Under light, the effect of GA_3_ was only observed at 20/7 °C and 25/10 °C (Figure 1).

- *Atropa belladonna*

GA_3_ only stimulated germination at 20/7 °C under light, but at 25/10 °C, 28/14 °C, and 32/18 °C under both light and dark, although the most significant increases occurred under darkness at 25/10 °C, 28/14 °C, and 32/18 °C, where only 5% of the untreated seeds germinated compared with 86% of the seeds treated with GA_3_ (Figure 1).

### 3.2. Outdoor Experiments

#### 3.2.1. Phenology of Seedling Emergence

- *Atropa baetica*

**Figure 3:**
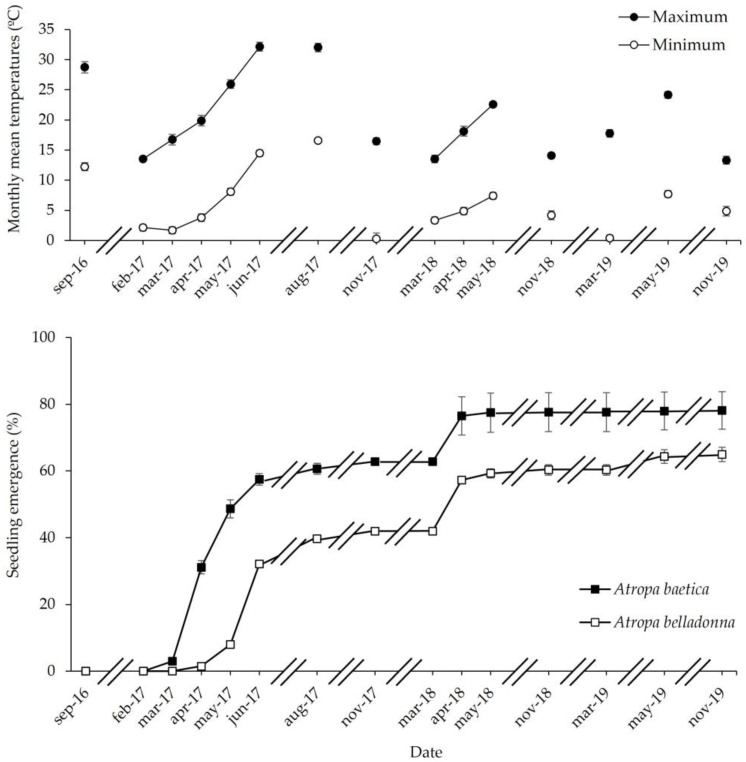
Phenology of cumulative seedling emergence in *Atropa baetica* and *Atropa belladonna* for 3 years in a shadehouse, and the maximum and minimum monthly mean temperatures recorded during the experiment.

No seedlings emerged between 1 September 2016 and 1 February 2017. Seedlings began to sprout (3%) on 1 March 2017 and the cumulative percentage increased throughout the following months: with 31% on 1 April, 48% on 1 May, and 58% on 1 June. Emergence then slowed down and only reached 61% on 1 September. During the second autumn post-sowing, seedling emergence changed little with 63% on 1 March 2018, but a new peak was recorded during the second late winter–early spring post-sowing, with 76% on 1 April 2018. Subsequently, almost no seedlings emerged, although some seedlings emerged at the beginning of the third spring post-sowing, where the cumulative percentage emergence was 78% on 1 November 2019 (Figure 3).

- *Atropa belladonna*

Seedlings did not emerge between 1 September 2016 and 1 March 2017, with only 1.5% on 1 April 2017. At the beginnings of the following months, the cumulative percentage emergence rates were 8% on 1 May, 32% on 1 June, and 40% on 1 August. During the second autumn post-sowing, the cumulative percentage emergence rate reached 42%. In addition, during the second late winter–early spring, a new emergence peak was recorded, with 57% on 1 April 2018. Finally, a new third peak occurred at the beginning of the third spring post-sowing, with 64% (Figure 3).

#### 3.2.2. Phenology of Dormancy Break and Radicle Emergence

- *Atropa baetica*

**Figure 4:**
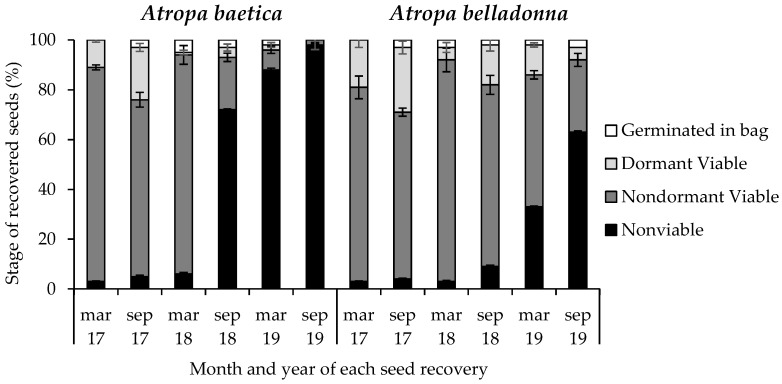
Changes under shadehouse conditions in the status of buried *Atropa baetica* and *Atropa belladonna* seeds after recovery every 6 months for 3 years.

Among the seeds recovered on 1 March 2017, 86% were viable nondormant, 11% were viable dormant, and 3% were nonviable. Among the seeds recovered on 1 September 2017, 71% were viable nondormant, 21% were viable dormant, 5% were nonviable, and 3% germinated in the bag. This result is important because 86% of the seeds were viable nondormant 6 months before. Among the seeds recovered subsequently, the percentage of seeds that germinated in the bag increased from a low level (0–5%), and most of the seeds were nonviable and approximately half of them were empty on the fourth seed recovery date (1 September 2018). On 1 September 2019, only 2% of the recovered seeds were viable (Figure 4).

- *Atropa belladonna*

Among the seeds recovered on 1 March 2017, 78% were viable nondormant, 19% were viable dormant, and 3% were nonviable. Among the seeds recovered on 1 September 2017, 67% were viable nondormant, 26% were viable dormant, 4% were nonviable, and 3% germinated in the bag. Among the seeds recovered subsequently, the percentage of seeds that germinated in the bag increased from a low level (2–3%) despite the fact that the seeds had previously overcome dormancy. It should be noted that 65% of the recovered *A. baetica* seeds were viable on 1 March 2019, and 34% on 1 September 2019. Thus, approximately half of the nonviable seeds were empty (Figure 4).

#### 3.2.3. Analysis of the Soil Seed Bank

Many of the seeds recovered from the soil seed bank were empty or nonviable, especially *A. baetica* seeds, but a considerable amount of the seeds were viable, with 294 viable *A. baetica* seeds/m^2^ and 262 viable *A. belladonna* seeds/m^2^. The ratio of nonviable seeds/viable seeds was 13.8 in *A. baetica* and 2.8 in *A. belladonna* (Table 1).

## 4. Discussion

The ripe seeds of *A. baetica* and *A. belladonna* had completely developed embryos and permeable coats, and they exhibited some level of physiological dormancy. Freshly matured seeds were characterized by conditional physiological dormancy, where they germinated at high temperatures (32/18 °C) but not at low and cool temperatures (5 °C, 15/4 °C, 20/7 °C). According to the results, the level of physiological dormancy was nondeep because germination was stimulated by: (1) short periods of dry seed storage, (2) GA_3_, and (3) short periods of cold stratification. Nondeep physiological dormancy is most frequent in vascular plants, and it includes five types [23], where type 2 is characterized by the minimum temperature for germination decreasing during the period of dormancy loss, as found for *A. baetica* and *A. belladonna* seeds.

In this study, the optimal temperature for germination was very high (32/18 °C) and warmer than the temperature recorded in the shadehouse in September (28/12 °C). Consequently, the optimal temperature for germination was an obstacle to seedling emergence in the autumn (Figure 1 and Figure 3). At seed dispersal time (seed age = 0 months), *A. baetica* and *A. belladonna* seeds did not germinate at 28/14 °C under dark, 25/10 °C under dark, 20/7 °C under dark, 15/4 °C under dark, and 5 °C under dark (Figure 1), and thus no seedling emergence occurred at the first autumn post-sowing (Figure 3). The increase in the germination capacity with both seed age and exposure to cold stratification (Figure 2) explains why the seedling emergence peak did not occur until the first period of winter ending–spring beginning following sowing, i.e., March–May for *A. baetica* and April–June for *A. belladonna*. In the shadehouse, the mean maximum and minimum temperatures in March and April were 16/2 °C and 18/4 °C, respectively. During the second autumn post-sowing, the cumulative emergence rate only increased by 2%, probably because the seeds that overcame dormancy during the previous months germinated if the temperature, humidity, and soil aeration conditions were appropriate. After the second winter post-sowing with cold stratification, a small new seedling emergence peak occurred at winter ending–spring beginning (Figure 3). This strategy reflects the adaptation of both species to their habitats because seedlings would not survive if they emerged at autumn ending–winter beginning. Our observations of these species have shown that seedlings can withstand occasional frosts, but they cannot survive repeated frosts. The high temperatures that are optimal for the germination of these species are not usual in Mediterranean areas, where many species germinate at low autumn temperatures (20/7 °C, 15/4 °C) [32]. However, some species require prior cold stratification before a low incubation temperature to obtain high germination rates, where this requirement hinders autumn germination and promotes radicle emergence in the spring to increase seedling survival. This pattern has been observed in other mid-mountain species that share habitats with the study species, such as *Delphinium fissum* subsp. *sordidum* [33] and *Aconitum napellus* subsp. *castellanum* [34].

The stimulation of germination by light in this study is an adaptation to the habitat of the target species because both species can colonize forest clearings and form persistent soil seed banks. Another feature that supports the capacity to grow in gaps between vegetation is germination at daily fluctuating temperatures of 15/4 °C, 20/7 °C, 25/10 °C, etc. [35]. The promotion of germination by light and fluctuating temperatures is common in herbaceous perennials that colonize forest clearings, such as *Stellaria graminea* and *Moehringia trinervia* [12].

Our analysis of the phenology of dormancy break showed that after 3 years, 98% and 66% of the *A. baetica* and *A. belladonna* seeds, respectively, were nonviable (Figure 4), which explained the low rate of seedling emergence during the third phenological cycle post-sowing. Only between 2% and 3% of seeds germinated in the bag in September 2017 despite the fact that most of the seeds were viable and nondormant 6 months before. The scarcity of seedlings could have been a consequence of the low germination rate under darkness conditions by *A. belladonna* seeds, although this did not apply to *A. baetica* seeds, which could not germinate due to the lack of oxygen inside the bags. Previous studies showed that factors such as low O_2_ concentrations [36] or high CO_2_ concentrations [37] prevented the germination of buried nondormant viable seeds.

The present study aimed to determine the relative importance of two strategies for germination regulation [14]: (1) a portion of seeds responded rapidly to environmental cues and germinated during the first phenological cycle post-sowing, and (2) other seeds germinated in the following cycles. In the case of *A. baetica*, 63% of the buried seeds germinated and became seedlings during the first cycle, and 15% of the seeds in the following cycles, whereas the remaining seeds probably lost their viability without contributing to germination regulation. Thus, the relationship between seeds sensitive to dormancy breakage/carryover seeds between years was 42/23 for *A. belladonna* and 63/15 for *A. baetica*, with a greater trend for *A. belladonna* seeds that remained in the soil (Figure 3 and Figure 4).

The seedling emergence rate was very low during the third phenological cycle, but the results indicated that both species could form persistent soil seed banks (Table 1). The numbers of viable recovered seeds were reduced, with 262 and 294 seeds/m^2^ for *A. belladonna* and *A. baetica*, respectively, but these values are very important in a population dynamics context because these species are unusual and the formation of persistent soil seeds banks can allow the recruitment of seedlings for years, especially when fruit formation might be affected by events such as herbivory or destruction by summer storms [18]. Similarly, population viability analyses have also stressed the important roles of soil seed banks in the long-term persistence of populations of perennials, such as *Helianthemum polygonoides*, which is a critically endangered perennial shrub in southeast Spain [38]. Vegetative propagation appears to be more important for seedling recruitment than seed germination for the study species, but seeds play a crucial role in the renewal of adult individuals, which can live more than 20 years (personal observation), although they inevitably die. In both species, the persistent soil seed banks were short-lived according to the nonviable/viable seed ratios of 2.8 for *A. belladonna* and 13.8 for *A. baetica*. Approximately half of the nonviable seeds recovered from soil samples were empty and they did not withstand slight pressure applied by tweezers when handled in Petri dishes at the start of the germination experiments. A similar proportion of empty seeds was found in the last bag recovered during our analysis of the phenology of dormancy. At the end of the experiment, most of the seeds were not viable after 3 years. Therefore, the duration of the persistent seed soil banks was determined as around 3–5 years.

**Figure 5:**
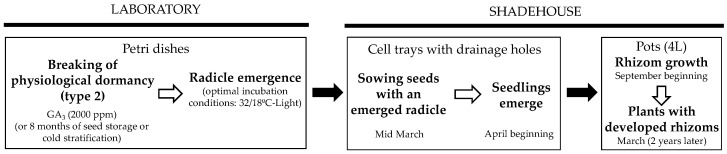
Protocol for plant production from seeds of *Atropa baetica* and *Atropa belladonna*.

A common protocol to produce plants from seeds has been elaborated for the two species (Figure 5). In general, *A. baetica* seeds had higher final germination percentages than *A. belladonna* seeds, and thus seed germination restrictions or problems should be rejected as an explanation for the scarcity of *A. baetica* populations, and the causes of its rarity may be related to other bottlenecks in its biological cycle. Therefore, future studies should focus on analyzing the recruitment and survival of new seedlings, or the establishment of new populations by seed-dispersing birds.

## 5. Conclusions

According to the results obtained in the present study, the seeds of both species were characterized by type 2 physiological dormancy and the optimal protocol established for producing plants from both seeds enables us to obtain plants without affecting the conservation of natural populations, or even with the aim of implementing reinforcement programs in their habitats.

## Figures and Tables

**Table 1:** Seed soil bank values (mean ± SE, *n* = 4) for natural populations of *Atropa baetica* and *Atropa belladonna*. Percentages of seeds in each state found in soil samples (20 × 20 × 5 cm).

Species	Nondormant Viable Seeds	Dormant Viable Seeds	Nonviable Seeds	Viable Seeds/m^2^
*Atropa baetica*	10 ± 3.16	1.75 ± 0.82	162.75 ± 51.71	293.75 ± 77.81
*Atropa belladonna*	9 ± 3.39	1.5 ± 1.11	29.5 ± 8.32	262.5 ± 89.26

## Data Availability

The data presented in this study are available on request from the corresponding author. The data are not publicly available due to privacy concerns.
